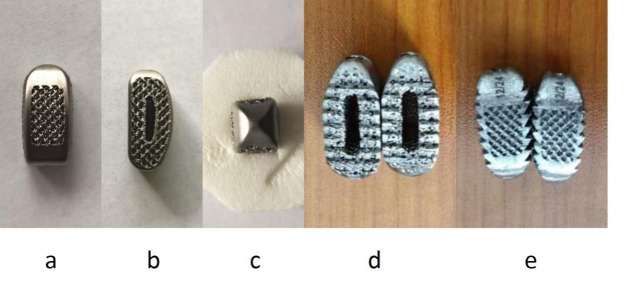# The postoperative clinical effects of utilizing 3D printed (Ti6Al4V) interbody fusion cages in posterior lumbar fusion: A retrospective cohort study

**DOI:** 10.1097/MD.0000000000038431

**Published:** 2024-06-21

**Authors:** Zi Wang, Dongzhe Zhang, Zepei Zhang, Jun Miao

**Affiliations:** a Department of Spine Surgery, Tianjin Hospital, Tianjin, China; b Department of Spine Surgery, Cangzhou Hospital of Integrated TCM-WM, Cangzhou, China.

**Keywords:** 3D printing, cage, degenerative disc disease, implants, increasing material manufacturing

## Abstract

**Background:**

The research focused on the postoperative effect of using interbody fusion cage in lumbar posterior lamina decompression and interbody fusion with pedicle screw by comparing the postoperative effect of using 3D printing (Ti6Al4V) and PEEK material interbody fusion cage.

**Methods:**

Ninety-one patients with lumbar degenerative diseases from the Department of Spine Surgery of Tianjin Hospital were included in the study cohort. They were divided into 3D group (n = 39) and PEEK group (n = 52) according to the use of interbody fusion cage. The imaging data of the patients were collected and the postoperative data of the 2 groups were compared to evaluate patients’ health status and the recovery of lumbar structure and function after operation.

**Results:**

Combined with the degree of fusion, the clinical effect of 3D printing titanium alloy interbody fusion cage was comprehensively judged. At the last follow-up, the JOA score, ODI index, VAS, prolo function score, and SF-36 scale of the 2 groups showed that the clinical symptoms were better than those before operation (*P* < .05). The height of intervertebral disc, the area of intervertebral foramen and the physiological curvature of lumbar vertebrae increased in varying degrees after operation (*P* < .05). At the last follow-up, the vertebral cage fusion rates were as high as 89.13% and 90.91% in the 3D and PEEK groups, with collapse rates of 6.5% and 4.5%, respectively. There were 10 cases of cage displacement in 3D group and 7 cases of cage displacement in PEEK group. There was no significant difference between the 2 groups (*P* > .05).

**Conclusions:**

In conclusion, 3D printed (Ti6Al4V) interbody fusion cage can obtain good clinical effect in the surgical treatment of lumbar degenerative diseases. Posterior lumbar lamina decompression, bilateral pedicle screw fixation combined with 3D printed cage interbody fusion is excellent in rebuilding the stability of lumbar vertebrae. 3D printed interbody fusion cage can be an ideal substitute material for intervertebral bone grafting. The stable fusion time of interbody fusion cage after lumbar fusion is mostly from 3 months to half a year after operation.

## 1. Introduction

Intervertebral disc degeneration is a pathophysiologic process in which the biochemical environment of the intervertebral disc cells is altered by a variety of factors, resulting in the gradual loss of function of the intervertebral disc. Patients often experience clinical manifestations such as neck and shoulder pain, lower back pain, and lumbar and leg pain, which can have a serious impact on the patient’s work and life.^[1]^ In lumbar vertebrae, L4/L5 segment has the highest probability of intervertebral disc degeneration. Age and obesity are the main causes of intervertebral disc degeneration.^[2]^ Conservative treatment includes ergonomic guidance, active physiotherapy, educational counseling and family exercise guidance, non-steroidal anti-inflammatory drugs^[3]^ and lumbosacral intervertebral foramen epidural drug injection guided by CT.^[4]^ As the gold standard surgery for lumbar degenerative diseases, discectomy and fusion has been proven to be effective in restoring the height and stability of the intervertebral space through extensive clinical practice.^[5]^ The surgical procedure of this study was posterior interbody fusion (PLIF), which is one of the spinal fusion procedures. Briggs and Milligan^[6]^ explained this procedure for the first time in history, using laminectomy and bone grafting in the intervertebral disc space. Jaslow^[7]^ updated the same procedure in 1946 to remove the spinous process in the intervertebral space. It was not until 1953 that Cloward^[8]^ used an autografted iliac bone block. This led to better improvements that led to the popularization of the PLIF procedure. Traditional interbody fusion usually takes the form of implanted bone tissue to stabilize the balance between the vertebrae, and autogenous bone has been shown to be excellent in terms of osteogenicity and mechanics.^[9]^ However, complications such as pain and infection in the donor area have led to a growing demand for bone graft substitutes. The advent of interbody fusion devices has facilitated the level of interbody fusion.

In recent years, the development of 3D printing technology and new printing materials make 3D printing interbody fusion cage possible and more and more used in clinic.^[10,11]^ 3D printing is also called “augmented manufacturing.” It begins with the use of medical image processing technology to create a model of the image data, and reverse engineering software to implement the three-dimensional model reconstruction, with the assistance of computer software, personalized design and reconstruction of the model. The data is then entered into a 3D printer with computer-aided manufacturing software. Finally, the three-dimensional entity with high precision, rapid prototyping and individualization is formed by the layer-by-layer accumulation of adhesive materials. A good interbody fusion cage needs to have an appropriate modulus of elasticity to provide sufficient hardness to ensure stability and to prevent sinking. In addition, biocompatibility is needed to reduce rejection and improve fusion rate. Sung-SooChung^[12]^ analyzed the postoperative curative effect of 40 patients with 3D printed interbody fusion cage in PLIF operation and obtained good clinical and imaging results. FulvioTartara^[13]^ also made a follow-up study on the sagittal correction and subsidence of 18 patients undergoing ALIF/XLIF surgery after using 3D printing cage. The results also reflect the clinical effect of 3D printing cage. As the application of spinal fusion in lumbar degenerative diseases, PLIF surgery has achieved good results. Combined with the fixation of posterior screw-rod system and the use of interbody fusion cage, PLIF surgery is of great help in restoring intervertebral space height, providing vertebral body stability and improving patients’ symptoms. The interbody fusion cages are usually manufactured by compression molding, using titanium mesh, PEEK, carbon fiber and other materials. The invention and manufacture of 3D printing interbody fusion cage brings a new direction for spinal fusion. KirkMcGilvray^[14]^ used the 3D printed interbody fusion cage made of porous titanium alloy in sheep and it can provide good fusion and stability. However, there are few studies on the clinical application of 3D printing porous titanium alloy interbody fusion cage. This study focuses on the clinical and imaging data of patients who used 3D printed interbody fusion cage after operation.

## 2. Methods

### 2.1. Study design, setting and population

In this study, 91 patients who met the inclusion criteria were analyzed retrospectively. The patients underwent posterior lumbar lamina decompression and pedicle screw fixation interbody fusion in the Department of Spinal surgery of Tianjin Hospital from October 2018 to December 2019. Among them, 39 patients were diagnosed as lumbar disc herniation, 48 patients with degenerative lumbar spinal stenosis and 4 patients with degenerative spondylolisthesis. The follow-up period was more than 6 months. According to the different use of interbody fusion cage, the patients were divided into 3D group (39 cases) and PEEK group (52 cases) (Table 1). The clinical efficacy was evaluated and recorded preoperative, immediate postoperative, 1 month after operation, 3 months after operation, half a year after operation and the last follow-up. In the postoperative follow-up, the curative effect data of the patients were collected in the form of questionnaire. The lumbar MRI, CT, and X-ray data of patients at each follow-up time were collected and the sagittal values including intervertebral disc height, intervertebral foraminal height, intervertebral foraminal area, lumbar lordosis angle, segmental lumbar lordosis angle and central ratio were measured to evaluate the operation-related data. The fusion effect of interbody fusion cage was analyzed by fusion rate.

**Table 1:** Patient demographics.

Group	Sex, male/female	Age (yr)	Follow-up times (mo)	PLIF level				
				L3/L4	L4/L5	L5/S1	L3/L4, L4/L5	L4/L5, L5/S1
3D	16/23	61.28 ± 9.06	16.74 ± 7.17	1	29	2	4	3
PEEK	18/34	61.52 ± 6.63	20.94 ± 10.89	0	32	6	10	4

PLIF = posterior lumbar interbody fusion.

The selection criteria were as follows: in accordance with the diagnosis of lumbar degenerative diseases, including lumbar disc herniation, degenerative lumbar spinal stenosis and degenerative spondylolisthesis; ineffective in conservative treatment, symptoms significantly or seriously affect work and life; operative segment ≤ 2; have undergone PLIF surgery; preoperative and postoperative imaging data and clinical efficacy evaluation data is complete. The exclusion criteria were as follows: previous history of spinal surgery; previous history of spinal tumor, ankylosing spondylitis and spinal tuberculosis; anterior or lateral approach; scoliosis with coronal lumbar cobb angle greater than 10°; no screw-rod system internal fixation or only unilateral internal fixation; and incomplete imaging data or failure to follow up in the outpatient clinic. The study was approved by the Institutional Review Board of Tianjin Hospital and was conducted in accordance with the Declaration of Helsinki. Because this study is a retrospective study, all patients are exempted from signing informed consent.

### 2.2. Introduction of 3D printed (Ti6Al4V) interbody fusion cage

In this study, the 3D printed (Ti6Al4V) cages were produced by Beijing Aikang Yicheng Medical equipment Company Limited. It has a similar rectangular design and the outer wall is curved. The inside and outside bevel is 8° angle. The front and rear diameter is 22 to 29 mm. The left and right diameters are 10 and 11 mm and the height is 7 to 15 mm. The head is prismatic bullet-shaped design and there is a hole in the tail (Figure S1, Supplemental Digital Content, http://links.lww.com/MD/M924).

### 2.3. Surgical technique

All the patients were performed with posterior lumbar interbody fusion (PLIF). And all the operations were performed by senior director who had been engaged in spinal surgery for a long time. Prone position was taken after successful anesthesia. The spinous process of lumbar operation segment corresponding to skin incision, about 5 to 15 cm long, bilateral subperiosteal peeling off the paraspinal muscle to the outer edge of the articular process. Four or 6 pedicle screws were screwed at the entry point of the pedicle of the corresponding segments on both sides. The whole plate decompression was performed on the operative segment. The intervertebral disc could be seen and the bilateral nerve root channels were released. Then removed the intervertebral disc. The height of intervertebral space was measured by test model and an appropriate amount of autogenous bone was implanted after washing intervertebral space. According to the test results, the appropriate height 3D printing or PEEK interbody fusion cage was selected and implanted into the intervertebral space. There was no obvious compression in each nerve channel after re-exploration. After sufficient hemostasis, 1 wound drainage tube was indwelled. The wound drainage tube was retained for 36 to 48 hours after operation. The brace was used to move under the ground after the drainage tube was pulled out and the lumbar vertebrae were examined by CT and X-ray. Antibiotics were used to prevent infection in 24 hours after operation.

### 2.4. Clinical evaluation

The indexes related to operation include operation time, blood loss and hospital stay. Indicators related to clinical efficacy include: We used the Japanese Orthopaedic Association Evaluation treatment score (JOA) to evaluate human functional disorders. The improvement rate of the post-treatment score, that is, (postoperative JOA score-preoperative JOA score)/(29-preoperative JOA score) * 100% to help evaluate the degree of improvement. Oswestry Disability Index was used to evaluate lumbar function. Pain visual analogue scale was used to evaluate the degree of pain. Prolo functional score (PFs) was used to evaluate the degree of postoperative recovery and health SF-36 scale was used to evaluate the overall health status of patients from 8 angles.

### 2.5. Radiological measurements

**Figure 1.**
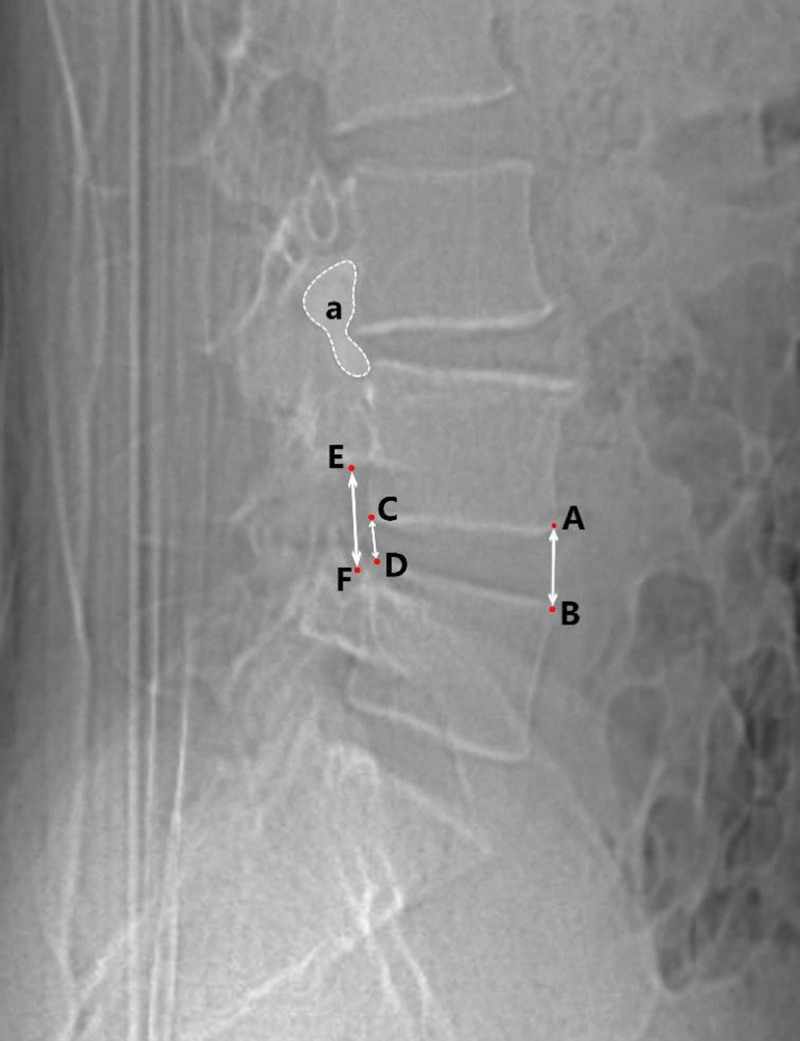
Lateral X-ray of lumbar vertebra. AB-height: anterior disc height. CD-height: posterior disc height. EF-height: foraminal height. a-area: foraminal area.

**Figure 2.**
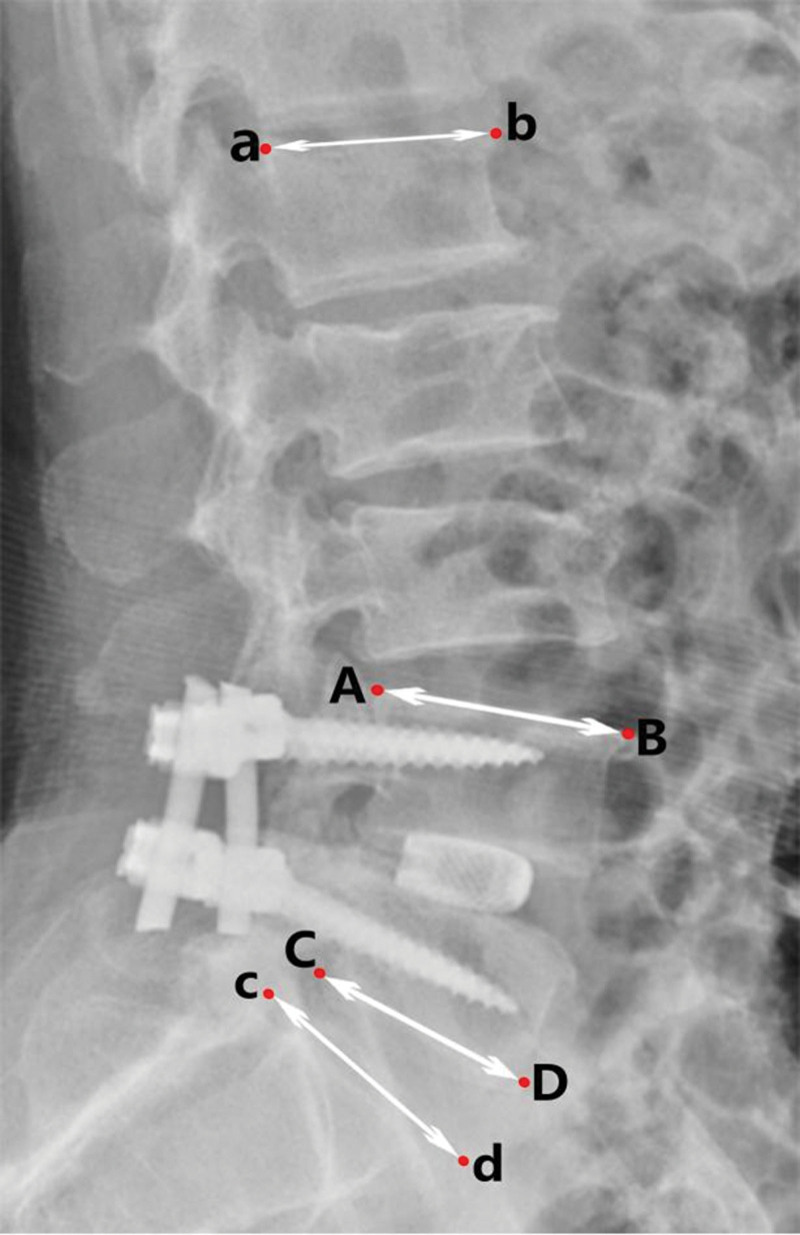
Lateral X-ray of lumbar vertebra. Segmental lumbar lordosis angle: the angle formed by the extension line of segment AB and segment CD. Lumbar lordosis angle: the angle formed by the extension line of segment ab and segment cd.

**Figure 3.**
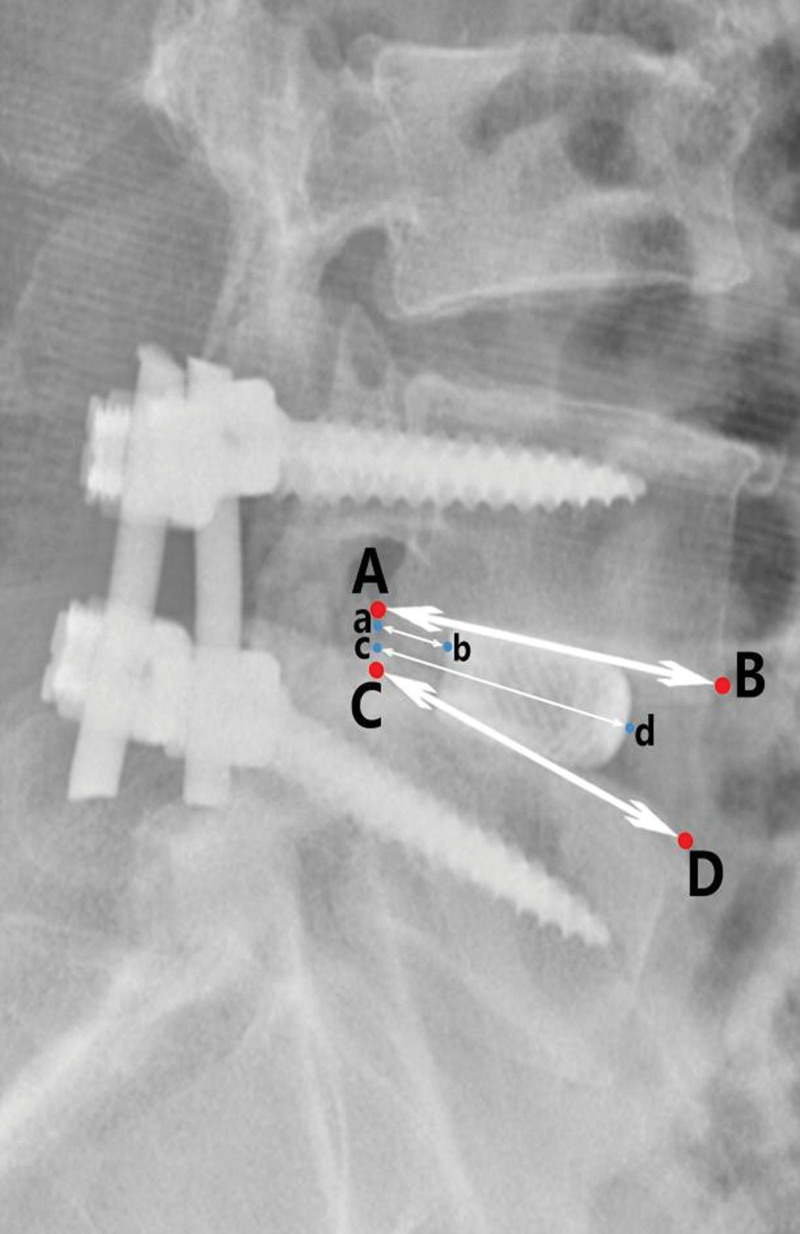
Lateral X-ray of lumbar vertebra. Central ratio = (ab + cd)/(AB + CD)*100%.

All patients underwent lumbar MRI, CT, and X-ray examination before operation and lumbar X-ray examination was performed immediately postoperative, 1 month after operation, 3 months after operation, half a year after operation and the last follow-up. Lumbar CT auxiliary examination was performed when X-ray examination was difficult to judge fusion. In each follow-up, the imaging data included intervertebral disc height, intervertebral foraminal height, intervertebral foraminal area, lumbar lordosis angle, segmental lumbar lordosis angle, cage central ratio and vertebral fusion rate. The judgment criteria of vertebral fusion were modified according to the criteria of Bridwell-Lenke.^[15]^ The degree of vertebral fusion was divided into three grades. Grade 1: graft intact and continuous trabecular formation between graft and vertebral endplate could be seen; grade 2: graft intact and no continuous trabecular formation between graft and vertebral endplate or clear transparent shadow at the top or bottom of graft; grade 3: no fusion and bone graft absorption and collapse. Grade 1 was identified as the fusion of vertebral body and cage. The image data were uploaded by the Imaging Department of Tianjin Hospital and measured manually by 2 spinal surgeons. The imaging data are measured as follows (Figs. 1–3):

1. Intervertebral disc height: the average height of the anterior and posterior edge of the intervertebral disc in the sagittal position.
2. Intervertebral foraminal height: the furthest distance between the lower edge of the superior vertebral pedicle and the superior edge of the lower vertebral pedicle in the sagittal position.
3. Intervertebral foraminal area: the area surrounded by the inferior edge of the pedicle of the superior vertebral body, the superior edge of the pedicle of the inferior vertebral body and the posterior edge of the vertebral body in the sagittal position.
4. Lumbar lordosis angle: the angle between the extension line of the superior endplate of L1 vertebral body and the superior endplate of S1 vertebral body.
5. Segmental lumbar lordosis angle: the angle between the extension line of the superior endplate of the superior vertebral body and the inferior endplate of the lower vertebral body.
6. Central ratio: the distance from the posterior edge of cage to the posterior edge of vertebral body (ab), the distance from the anterior edge of cage to the posterior edge of vertebral body (cd), the length of inferior endplate of upper vertebral body (AB) and the length of superior endplate of lower vertebral body (CD) were measured. Central ratio = (ab + cd)/ (AB + CD) * 100%.

### 2.6. Statistical analysis

Statistical analyses were performed with SPSS version 27.0 (IBM SPSS statistics, Chicago). The continuous variables were presented as mean ± standard deviation. The preoperative and postoperative data in the same group were analyzed by paired sample *t* test. The similar data between different groups were compared by independent sample *t* test and the fusion rates of the 2 groups were compared by chi-square test. The derived *P* < .05 were considered statistically significant.

## 3. Results

### 3.1. Operation related information

A total of 91 cases were included in this study. 32 cases of 3D group and 38 cases of PEEK group had no significant difference in operation time, blood loss and hospital stay. Similarly, there was no significant difference in these data between 7 cases of 3D double-segment group and 14 cases of PEEK double-segment group (Table 2). There were 46 operative segments in 39 cases in 3D group and the average height of cage was 8.87 ± 1.19 mm. There were 66 operative segments in 52 cases in PEEK group and the average height of cage was 9.33 ± 1.34 mm. The data accords with normal distribution and has homogeneity of variance which is analyzed by independent sample *t* test (*P* = .06). There was no significant difference in cage height between the 2 groups.

**Table 2:** Operation related information.

Characteristics	Group	Number	Operation time (min)	Blood loss (mL)	Hospitalization time (d)
Single segment	3D	32	136.09 ± 37.82	261.56 ± 104.97	10.47 ± 3.09
	PEEK	38	158.16 ± 55.73	339.47 ± 220.31	10.71 ± 3.64
Double segments	3D	7	185.00 ± 38.08	485.71 ± 211.57	9.14 ± 2.79
	PEEK	14	192.5 ± 34.12	592.85 ± 301.83	11.14 ± 2.84

### 3.2. Clinical outcomes

**Figure 4.**
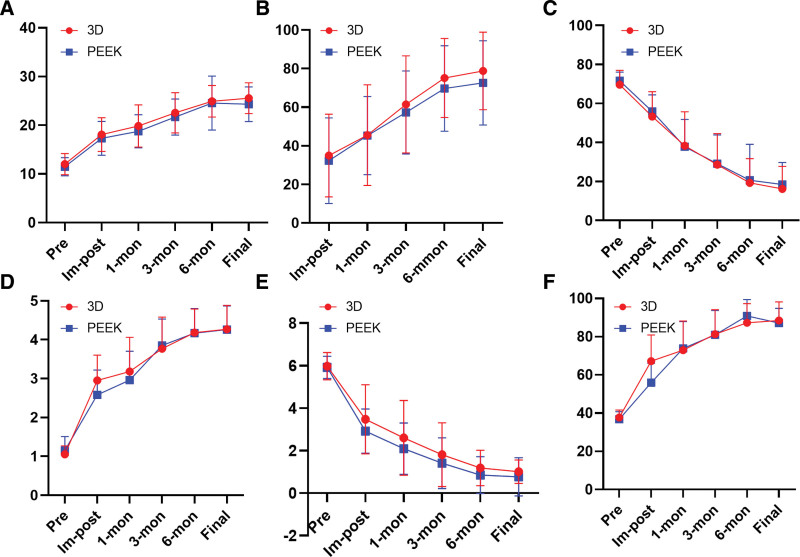
Clinical outcomes of 3D group and PEEK group at preoperative (Pre), immediate postoperative (Im-post), 1-month (1-mon), 3-month (3-mon), 6-month (6-mon), and final follow-up (Final) after surgery. (A) Changes of JOA score. (B) Changes of JOA improvement rate. (C) Changes of ODI index. (D) Changes of PFs. (E) Changes of VAS. (F) Changes of SF-36 score. JOA = The Japanese Orthopaedic Association Evaluation treatment score, ODI = Oswestry Disability Index, PFs = prolo function score.

The JOA, the improvement rate of the post-treatment score, Oswestry Disability Index, prolo functional score, visual analogue scale and health SF-36 scale scores were recorded preoperative, immediate postoperative, 1 month after operation, 3 months after operation, half a year after operation and the last follow-up. The scores showed that the clinical effects of the 2 groups were better than those before operation (*P* < .05) and showed a trend of gradual improvement in each stage after operation. There was no significant difference between the two groups. The changing trend of each score is shown in Figure 4.

### 3.3. Radiological measurements

**Figure 5.**
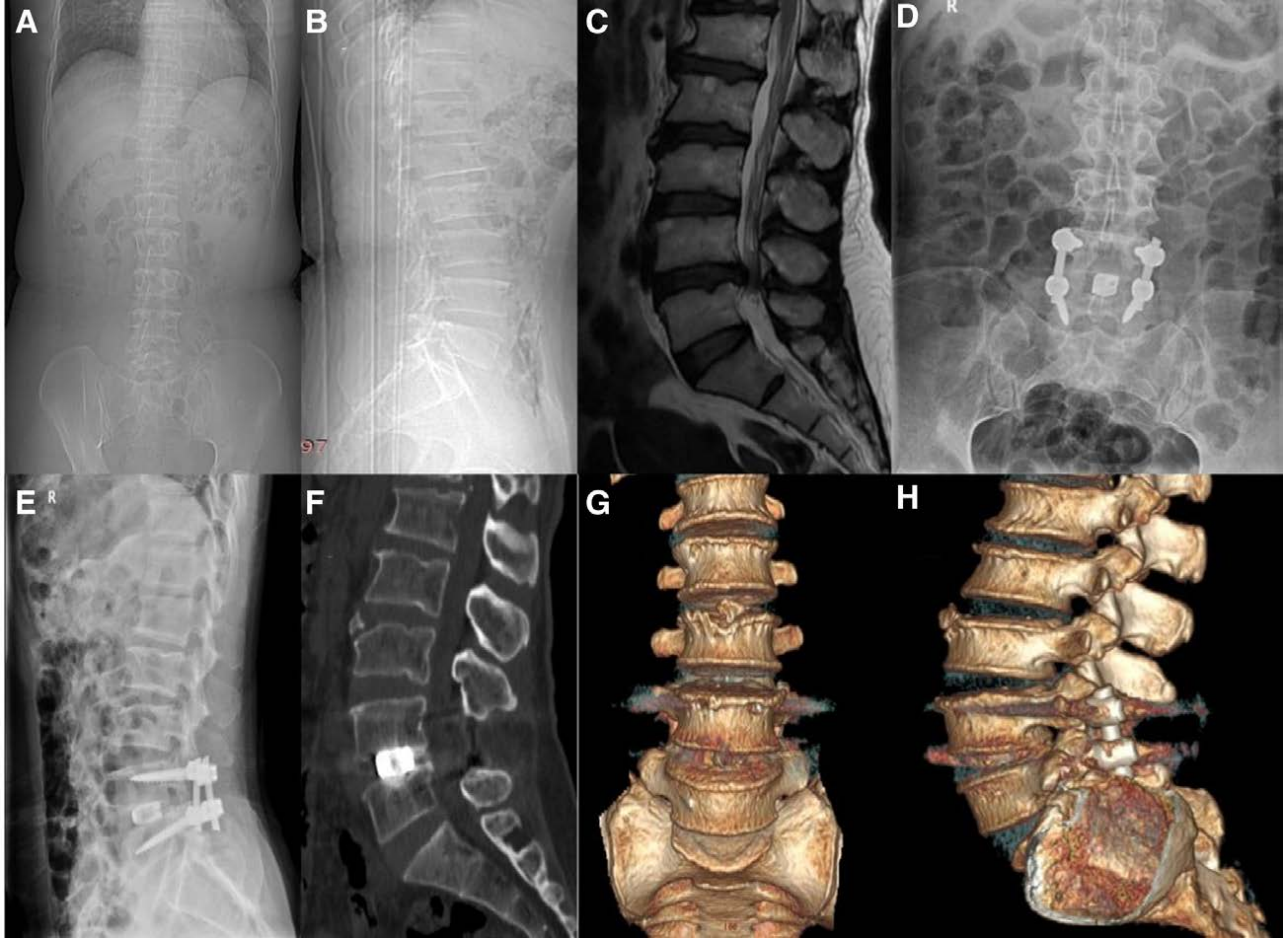
A 51-year-old female patient diagnosed as lumbar spinal stenosis. We evaluated her preoperative clinical outcome. The JOA score was 13, the ODI index was 66%, the PFs was 1, the VAS was 6 and the SF-36 score was 42.5%. And here were her radiographic measurements before surgery. The intervertebral disc height was 9.45 mm, the foraminal height was 16.5 mm, the foraminal area was 108.7 mm², the segmental lumbar lordosis angle was 18.7°, the lumbar lordosis angle was 36.5°. We measured the radiographic data immediately after the PLIF surgery on L4/L5. The intervertebral disc height was 11.8 mm, the foraminal height was 17.9 mm, the foraminal area was 115.4 mm², the segmental lumbar lordosis angle was 20.5°, the lumbar lordosis angle was 42.4°. We evaluated her clinical outcome at 6-month postoperative. The JOA score was 27, the ODI index was 10%, the PFs was 4, the VAS was 0 and the SF-36 score was 92.5%. (A and B) Anterior and lateral X-ray of lumbar spine before surgery. (C) Lumbar MRI before surgery. (D and E) 6-month postoperative anterior and lateral X-ray of lumbar spine. (F–H) 6-month postoperative lumbar CT and three-dimensional reconstruction. JOA = The Japanese Orthopaedic Association Evaluation treatment score, ODI = Oswestry Disability Index, PFs = prolo function score, PLIF = posterior lumbar interbody fusion, VAS = pain visual analogue score.

There were 39 cases (32 cases of single segment and 7 cases of double segments) in 3D group including 5 cases of L3/L4 segment, 36 cases of L4/L5 segment and 5 cases of L5/S1 segment. There were 52 cases (38 cases of single segment and 14 cases of double segments) in PEEK group including 10 cases of L3/L4 segment, 46 cases of L4/L5 segment and 10 cases of L5/S1 segment. The imaging data will be compared and analyzed according to different segments. The data of intervertebral disc height showed that in L3/L4 segment, the last follow-up in 3D group increased by 1.38 ± 1.06 mm compared with that before operation (*P* < .05). In L4/L5 segment, the immediate and last follow-up of 3D group increased by 1.53 ± 1.42 mm (*P* < .05) and 1.39 ± 2.01 mm (*P* < .05) while that of PEEK group increased by 1.40 ± 1.23 mm (*P* < .05) and 0.96 ± 1.33 mm (*P* < .05). In L5/S1 segment, the last follow-up in 3D group increased by 3.43 ± 1.56 mm compared with that before operation (*P* < .05). It is worth noting that at the last follow-up, the intervertebral disc height of L5/S1 segment in 3D group was higher than that in PEEK group (*P* < .05) (Table 3). The height of intervertebral foramen in each operative segment of the two groups increased after operation, but the change was not obvious (Table 3). After measuring the area of intervertebral foramen, we found that in L3/L4 segment the last follow-up increased by 34.56 ± 26.96 mm^2^ (*P* < .05) in 3D group and 56.41 ± 65.65 mm^2^ (*P* < .05) in PEEK group and there was no significant difference between the 2 groups. In L4/L5 segment, the immediate and last follow-up of 3D group increased by 10.39 ± 26.45 mm^2^ (*P* < .05) and 7.71 ± 37.85 mm^2^ (*P* > .05) while that of PEEK group increased by 13.02 ± 33.61 mm^2^ (*P* < .05) and 7.86 ± 46.34 mm^2^ (*P* > .05). In L5/S1 segment, the last follow-up increased by 28.53 ± 22.13 mm^2^ (*P* < .05) in 3D group and 7.71 ± 33.26 mm^2^ (*P* > .05) in PEEK group and there was no significant difference between the 2 groups (Table 3). The results of segmental lumbar lordosis angle showed that in L4/L5 segment, the immediate and last follow-up of 3D group increased by 2.29 ± 5.58° (*P* < .05) and 1.91 ± 5.12° (*P* < .05) while that of PEEK group increased by 2.24 ± 4.95° (*P* < .05) and 2.50 ± 4.68° (*P* < .05). In L5/S1 segment, 3D group increased 3.88 ± 6.12° (*P* > .05) immediately after operation while PEEK group increased 8.18 ± 6.37° (*P* < .05). The added value of PEEK group is higher than that of 3D group (*P* < .05; Table 3). The lumbar lordosis angles of the 2 groups were statistically compared according to single and double segments (Table 4). The central ratio of cage represents the position of cage in the superior sagittal intervertebral space. The higher the value, the farther the cage is from the spinal canal. A total of 46 cages in the 3D group decreased by 3.63 ± 9.64% (*P* < .05) and 2.26 ± 8.01% (*P* < .05) at 1 month and the last follow-up while 66 cages in the PEEK group decreased by 1.17 ± 3.65% and 1.24 ± 4.51% at 1 month and the last follow-up (Table 4). We evaluated the fusion degree of cage and vertebral body in each segment of the 2 groups. The grade 1 was called fusion. The fusion rates of the 3D group were 8.70%, 76.10%, and 89.13% and the fusion rates of the PEEK group were 16.67%, 68.18%, and 90.91%. Chi-square test was used to analyze the fusion results of the last follow-up of the 2 groups (*P* = .756), the difference was not statistically significant (Table 5). During the operation and follow-up, no nail and rod breakage, vertebral compression fracture, immune rejection, pseudarthrosis, pulmonary embolism and intraoperative macrovascular injury were found in both groups. The main complications included nerve root injury, intraoperative hemorrhage (more than 700 mL), cerebrospinal fluid leakage, wound infection, cage displacement and cage collapse. A typical case is introduced here (Fig. 5).

**Table 3:** Preoperative and postoperative intervertebral disc height (mm).

Position	Clinical outcomes	Group	Preoperative	Im-post	1-month	3-month	6-month	Final
L3/L4	Intervertebral disc height (mm)	3D	7.31 ± 0.83	8.04 ± 0.89	9.13 ± 0.30*	9.33 ± 0.98*	9.21 ± 1.09*	8.69 ± 0.69*
		PEEK	8.46 ± 1.55	9.14 ± 1.62	9.83 ± 2.19	10.15 ± 1.86	9.97 ± 2.53	9.47 ± 1.33
	Foraminal height (mm)	3D	19.90 ± 1.04	20.15 ± 2.95	22.33 ± 4.00	22.11 ± 1.15	21.64 ± 2.09	21.33 ± 2.34
		PEEK	20.39 ± 1.42	20.65 ± 2.25	22.00 ± 3.98	22.23 ± 3.02	22.19 ± 1.93	21.58 ± 2.78
	Foraminal area (mm²)	3D	175.44 ± 25.06	199.53 ± 25.74*	220.71 ± 45.69*	198.29 ± 9.58*	202.36 ± 14.04*	210.00 ± 25.53*
		PEEK	158.36 ± 37.20	206.42 ± 46.02*	216.62 ± 51.29*	215.96 ± 39.74*	219.88 ± 43.41*	214.76 ± 46.38*
	Lumbar lordosis angle (°)	3D	6.44 ± 5.04	7.20 ± 3.61	7.18 ± 2.04	8.20 ± 4.32	8.50 ± 3.25	9.01 ± 4.36
		PEEK	8.73 ± 6.06	9.72 ± 5.43	8.67 ± 5.54	9.03 ± 5.64	8.38 ± 4.7	9.24 ± 5.42
L4/L5	Intervertebral disc height (mm)	3D	8.27 ± 1.77	9.8 ± 1.78*	10.60 ± 1.67*	10.28 ± 1.36*	9.92 ± 1.46*	9.66 ± 1.72*
		PEEK	8.19 ± 1.99	9.60 ± 2.04*	9.70 ± 1.91*	9.39 ± 1.59*	9.36 ± 1.79*	9.15 ± 1.63*
	Foraminal height (mm)	3D	18.04 ± 3.29	18.36 ± 2.57	18.91 ± 2.30	18.76 ± 2.15	18.57 ± 2.27	18.90 ± 2.16
		PEEK	18.85 ± 2.19	19.22 ± 2.53	19.75 ± 2.61	19.58 ± 2.23	19.24 ± 2.52	19.10 ± 2.06
	Foraminal area (mm²)	3D	147.16 ± 39.18	157.55 ± 39.02*	154.45 ± 46.64	153.31 ± 42.95	158.25 ± 32.67	154.87 ± 23.51
		PEEK	153.94 ± 38.27	166.96 ± 37.79*	166.17 ± 36.03*	163.57 ± 36.38	163.11 ± 34.23	161.80 ± 33.15
	Lumbar lordosis angle (°)	3D	16.75 ± 7.94	19.04 ± 6.61*	18.18 ± 6.08*	18.59 ± 6.10*	18.67 ± 5.69*	18.67 ± 6.52*
		PEEK	15.11 ± 7.05	17.35 ± 5.56*	17.44 ± 6.65*	17.77 ± 6.38*	17.40 ± 6.48*	17.61 ± 6.72*
L5/S1	Intervertebral disc height (mm)	3D	8.00 ± 3.69	10.32 ± 2.91	9.97 ± 2.99	11.25 ± 2.48*	10.84 ± 2.57*	11.43 ± 3.55*
		PEEK	8.31 ± 1.63	8.81 ± 1.84	9.55 ± 1.94	8.77 ± 1.54†	8.84 ± 1.66†	8.81 ± 1.50†
	Foraminal height (mm)	3D	14.18 ± 3.18	15.20 ± 2.81	13.89 ± 3.20	15.40 ± 1.40	14.51 ± 1.46	15.03 ± 1.94
		PEEK	16.35 ± 2.65	16.46 ± 2.16	16.74 ± 3.72	16.58 ± 2.57	17.15 ± 2.33	16.92 ± 2.38
	Foraminal area (mm²)	3D	98.05 ± 45.17	130.72 ± 54.28*	117.68 ± 41.34*	109.25 ± 33.68*	114.06 ± 36.42*	126.58 ± 52.82*
		PEEK	126.38 ± 52.31	134.96 ± 27.94	135.99 ± 47.03	133.22 ± 33.81	133.46 ± 33.79	129.64 ± 35.53
	Lumbar lordosis angle (°)	3D	13.00 ± 8.15	16.88 ± 3.19	15.02 ± 5.90	15.48 ± 4.61	19.38 ± 1.99	18.16 ± 5.46*
		PEEK	17.48 ± 7.73	25.66 ± 6.69*†	23.14 ± 6.47	22.38 ± 8.22	20.36 ± 7.59	20.33 ± 7.37

* Significance (*P* < .05) at postoperative intervals when compared with the preoperative data.

† Significance (*P* < .05) at postoperative intervals when compared with the 3D group at same time and segment data.

**Table 4:** Preoperative and postoperative lumbar lordosis angle and central ratio of cages.

Characteristics	Group	preoperative	Im-post	1-month	3-month	6-month	Final
Single segment (°)	3D	40.37 ± 13.06	42.03 ± 10.16	40.45 ± 10.06	40.82 ± 10.69	41.66 ± 11.22	43.35 ± 11.58
	PEEK	40.73 ± 15.38	42.89 ± 12.86	40.58 ± 13.23	41.41 ± 14.17	43.11 ± 13.45	43.03 ± 13.77
Double segments (°)	3D	29.43 ± 16.48	35.36 ± 10.32	32.04 ± 9.49	35.33 ± 10.35	35.96 ± 7.07	36.54 ± 8.32
	PEEK	42.37 ± 14.11	46.48 ± 8.90	45.13 ± 12.36	43.73 ± 11.17	43.80 ± 10.47	45.44 ± 11.58
Central ratio of cages (%)	3D		56.14 ± 6.71	52.48 ± 12.02*	51.39 ± 13.15*	53.65 ± 8.93*	53.87 ± 9.32*
	PEEK		55.62 ± 5.69	54.42 ± 6.93*	54.16 ± 6.69*	54.05 ± 7.03*	54.34 ± 7.54*

* Significance (*P* < .05) at postoperative intervals when compared with the immediate postoperative data.

**Table 5:** Postoperative fusion grade.

Group	3-month			6-month			Final		
	Grade 1	Grade 2	Grade 3	Grade 1	Grade 2	Grade 3	Grade 1	Grade 2	Grade 3
3D	4	39	3	35	8	3	41	5	0
PEEK	11	52	3	45	18	3	60	6	0

## 4. Discussion

Intervertebral disc, as the main weight-bearing tissue, plays a major role in maintaining the stability and function of lumbar spine.^[16]^ The interbody fusion material used in this study is titanium alloy Ti6Al4V which is the most common orthopedic titanium alloy material. The material is printed in 3D by electron beam melting technology. It has the advantages of less impurities and high-quality properties of metal. The finished 3D printed cage has a trabecular structure similar to that of bone: 80% porosity and 800 ± 200 μm pore size are beneficial to the migration and proliferation of osteocytes. Pore design allows cage to have an appropriate elastic modulus which makes cage not only have the advantages of strong osseointegration, good biocompatibility and high corrosion resistance, but also reduce the probability of postoperative cage collapse.

There was no significant difference in the height of cage between 2 groups. It was found that the 3D group had a slight advantage in operation time and blood loss. The reason may be that the 3D group has better preoperative preparation and smoother operation during the operation. The comprehensive evaluation scale showed that the patients were greatly improved immediately after operation and during the follow-up time and there was no significant difference between the 2 groups (*P* > .05). However, we found that the score of some patients at half a year or the last follow-up was lower than that immediately after operation which indicated that there were problems in postoperative interbody fusion and surgical trauma recovery. It required us to analyze the causes combined with imaging data.

When the height of the intervertebral disc returns to the normal value and gets stable fusion, the range of motion and stress capacity of the lumbar spine can be better restored. In addition, we analyzed the Pearson correlation between the intervertebral disc height and the intervertebral foraminal height. It found that there was a strong positive correlation between them, that is, with the recovery of the intervertebral disc height, the intervertebral foramen height will increase accordingly. It expands the scope of the nerve root outlet channel and makes the nerve root get better decompression. Cinotti et al^[17]^ reached the same conclusion after studying the correlation between intervertebral disc height and foramen stenosis. During this follow-up, we compared the intervertebral disc height of different segments between the 2 groups and found that: it was increased at the postoperative follow-up time compared with that pre-operation, especially in the L4/L5 segment; the intervertebral disc height of each segment in two groups at the last follow-up was lower than that at 3 months after operation, which was considered to be related to the fusion and absorption of interbody fusion cage, bone graft and vertebral body; and 3D group achieved better intervertebral disc height recovery than PEEK group at the last follow-up of L5/S1 segment. Intervertebral foramen as the outlet of nerve root will also affect the clinical performance of patients. Too small or too narrow intervertebral foramen will oppress the nerve root. We found that the height and area of intervertebral foramen increased to a certain extent at each time point after operation. The normal lumbar vertebrae show the shape of lordosis and a certain degree of lordosis angle is beneficial to the movement of lumbar vertebrae and the stability of weight bearing.^[18,19]^ The convex curve will cause serious instability and a series of clinical symptoms when it becomes straight or even inverted. Similarly, the excessive lordosis angle can also cause instability. Through this measurement, it was found that: the segmental lumbar lordosis angle of the two groups after operation was higher than that before operation, especially in L4/L5 segment (*P* < .05); and the increase of segmental lumbar lordosis angle in L5/S1 in PEEK group was more than that in 3D group (*P* < .05). It was found that when the lumbar lordosis angle is more than 50° or even 60° before operation, the lumbar lordosis angle decreases immediately after operation. It shows that PLIF surgery can restore the lumbar lordosis angle to some extent. But when the lordosis angle is too large, the operation can also correct the lumbar lordosis angle. Some studies^[20]^ had shown that the lumbar lordosis angle of normal adults is 47.46 ± 9.27° and it decreases when lumbar degenerative disease occurs, which is similar to our study. We introduce the concept of central ratio in order to describe the position of cage between vertebrae. That is the ratio of the distance between the central point of cage and the midpoint of the posterior edge of the upper and lower vertebral body to the average length of the upper and lower lamina in the sagittal plane. The higher the value, the farther the cage position is from the spinal canal. We can judge whether the cage has been shifted or not by measuring and calculating the central ratio at each follow-up time and then judging the fusion rate. We define a change in the central ratio of 5% to 10% as potential shift and a change of more than 10% or significant rotational shift as significant shift. In 3D group, the posterior significant shift of 5 cages occurred in 5 patients (10.9%). 2 cases were found that the cages shifted backward at 3 months after operation and protruded into the spinal canal and then underwent operation again. It is worth noting that the imaging data of these two patients immediately after operation showed that the central ratio of cages were 40.59% and 44.46% which was significantly less than the average of 56.14%. Potential shift of 5 cages occurred in 5 patients in 3D group (10.9%) at 1 month after operation. In the PEEK group, 1 cage was significantly shifted backward (1.5%) without protruding into the spinal canal and continued follow-up observation. Potential shift of 6 cages occurred in 6 patients in PEEK group (9.1%) at 1 month after operation. To sum up: the fusion between the vertebral body and the interbody fusion cage is not stable within 1 month after spinal fusion. The movement of the interbody fusion cage may be caused by some internal (osteoporosis, etc.) or external reasons (premature rehabilitation exercise, etc.). In severe cases, it will protrude into the spinal canal, which has a certain significance for postoperative rehabilitation guidance; most of the postoperative interbody fusion cage moves backward, so we can choose to place it in the middle and front to prevent the postoperative interbody fusion cage from moving backward and protruding into the spinal canal; and good compression fixation of pedicle screw system and perfect matching between fusion cage and endplate are helpful to reduce the possibility of displacement. Pan et al^[21]^ suggested that the possible factors affecting cage shift include bone mineral density, type of cage, surgical segment, endplate condition, height of intervertebral space and so on. Abbushi et al ^[22]^ believe that the placement of cage near the center of the intervertebral space will reduce cage displacement. Fusion rate is an important index to evaluate the effect of spinal fusion. In this study, the PEEK group achieved better results in preventing cage displacement which may be due to the fact that the upper and lower surface of the 3D printed cage was not rough enough, the surface friction was small and the position of cage placed close to the spinal canal during operation may lead to displacement. In the 3D group, there were 3 cases of cages protruding into the spinal canal. We modified the 3D printed data and made the upper and lower surface of cage to be zigzag to increase the surface friction after analyzing the reasons. The clinical effect needs further follow-up study. In addition, improper rehabilitation exercise after operation is also one of the reasons leading to cage displacement. At the last follow-up, the fusion rate of 3D group and PEEK group was 89.13% and 90.91% and there was no significant difference between the two groups. Similar to our results, Chung et al^[12]^ followed 40 patients with 53 segments of 3D printed titanium alloy interbody fusion cage. At the last follow-up, sagittal CT showed that the anterior bone bridge integrity rate of cage was 94.3% and the posterior bone bridge integrity rate was 86.7%. Good spinal fusion increases local stability and improving clinical symptoms. Although studies had shown that the correlation between imaging and clinical results is low.^[23,24]^ Good spinal fusion may not achieve better clinical results. However, there is also a contrary conclusion. Soini^[25]^ believed that good fusion will have better clinical results than patients with nonunion. In this study, we found that both groups achieved a good degree of fusion at the last follow-up and most of the fusion time was about half a year after operation. Early improper functional exercise, malnutrition and serious osteoporosis will affect the effect of early postoperative fusion.

During the follow-up, it was found that the main complications included nerve root injury, intraoperative hemorrhage (more than 700 mL), cerebrospinal fluid leakage, wound infection, cage displacement and cage collapse. In 3D group, 1 patient (2.6%) had new numbness in the right leg at 2 months after operation and the numbness disappeared half a year after operation. The intraoperative blood loss reached 700 mL in 1 case (2.6%). Cage was significantly shifted backward in 5 cases (10.9%). 2 patients (4.3%, all L4/L5 segments) had cage collapse immediately after operation and 1 patient (2.2%, L4/L5 segment) found cage collapse during follow-up 1 month after operation. In PEEK group, 3 patients (5.8%) had complications of postoperative nerve root injury, 1 patient (1.9%) had postoperative cerebrospinal fluid leakage. 1 patient (1.9%) developed wound infection one week after discharge and was re-admitted for wound debridement and anti-infective treatment. 5 patients (9.6%) had massive hemorrhage during operation (all more than 800 mL). 2 patients (3.0%, all L4/L5 segments) had cage collapse immediately after operation, and 1 case (1.5%, L4/L5 segment) 3 months after operation. Despite the possibility of intraoperative or postoperative complications, spinal fusion is still widely used in the field of spinal surgery because of its sufficient decompression, firm fusion and definite curative effect.^[26]^ There were fewer cases of intraoperative massive hemorrhage in 3D group (2.6%) than in PEEK group (9.6%), which may be due to better preoperative preparation and smoother intraoperative operation in 3D group. Corso^[27]^ calculated the postoperative revision rate of using 3D printed and PEEK interbody fusion cage. The results showed that 3D group was significantly better than PEEK group. We defined the 2 mm of the fusion cage protruding into the endplate as cage collapse. During the follow-up, there were 3 cases of cages collapse in both groups which indicated that the elastic modulus of cage was not consistent with the bone mineral density of the patients. It provided a consideration for the personalized design of 3D printed interbody fusion cages for different patients in the future. In addition, the possible causes of collapse are cage location, endplate factors, bone mineral density, cage material and design, intervertebral space overstretching and so on.^[28]^

As a new orthopedic implant in recent years, 3D printed interbody fusion cage has been paid more and more attention because of its unique advantages. 3D printing technology can increase the contact area between the fusion cage and the endplate. So it can help reduce the contact stress.^[29]^ Wu et al^[30]^ proved that the fusion cage with larger axial area can reduce the stress of the internal fixation system and the stress at the interface between the fusion cage and the end plate through finite element analysis. It plays a certain role in preventing the sinking of the fusion cage and the failure of the internal fixation system. In this study, the 3D printed interbody fusion cage is compared with the widely used PEEK material interbody fusion cage and we found some problems worthy of attention: The 3D printed cage can be improved in shape design including the design of zigzag surface to improve surface friction. We can design new 3D printed cages with special porosity to reduce the probability of postoperative collapse for the patients with different bone mass. The fusion cage will shift backward in a short time after operation, so it can be considered to place cage in the middle and anterior part of intervertebral space during operation. On the one hand it can help to improve the lumbar lordosis angle, on the other hand it can prevent cage from moving backward and protruding into the spinal canal. Some studies have suggested that the greater the amount of bone graft in PLIF surgery, the greater the probability of bone graft fusion.^[31]^ During the follow-up of the two groups of cases, we also have a better understanding of posterior lumbar interbody fusion: the appropriate cage height can be determined in combination with imaging data before operation. Adequate preoperative preparation can reduce the time of operation and then help to reduce the amount of intraoperative bleeding; measure the lumbar lordosis angle carefully and determine the degree of intraoperative recovery can complete the operation more effectively; and the preparation of the endplate before bone grafting is particularly important. Full scraping of the intervertebral disc tissue and cartilage endplate can make the cage and the endplate fit better and improve the postoperative fusion rate.

3D printing technology has been well applied in all aspects of orthopedics because of its advantage of personalized design. In spinal surgery, 3D printed cages has not only been used in lumbar surgery. Wu et al^[32]^ followed up 65 patients who used 3D printed interbody fusion cage after anterior cervical surgery. 3D group achieved better results in clinical efficacy and imaging data by compared with autogenous bone transplantation. Liu et al^[33]^ developed a biodegradable cage containing polycaprolactone and β-tricalcium phosphate which has been used in clinical lumbar surgery and achieved good results in follow-up. Li et al^[34]^ achieved the purpose of preventing surgical infection by adding vancomycin coating to 3D printing cage. Now, with the continuous update and progress of treatment concept and operation technology, different treatment methods will certainly bring better curative effect and fewer complications for patients. Currently, the use of 3D printing materials in orthopedics has proved to be widely superior.^[35]^ Dong and Pei^[36]^ et al concluded that the problems specific to 3D printing materials are mainly technical and physician awareness. As the production of 3D printed materials is mainly carried out by engineers, physicians familiar with the technology have lower seniority and are also less aware of the needs of orthopedic surgery, resulting in poor interaction between doctors and engineers. As a result, some of the models are just a complete reproduction of the image data and do not meet the needs of clinical applications. Similarly, some physicians blindly use 3D printing materials without adequate knowledge, resulting in the lack of good application of 3D printing materials. Despite the high cost of 3D printing, reducing the length of hospitalization to reduce complications can likewise reduce healthcare expenditures.^[37]^ At the same time, low-cost 3D printing materials are being extensively researched.^[38]^ Therefore, the promotion of 3D printing materials is more to raise the awareness of doctors and patients and increase the communication of medical-industrial interaction.

Our study has some limitations. First, the sample size was small due to the small number of patients using 3D printed materials. However, we believe that these data will provide valuable information for the future application of 3D printing materials, and further expansion of the sample is needed to validate the safety and efficacy of 3D printing materials. Second, this was a retrospective study with a short follow-up period of 6 months. However, the average duration of osseointegration is approximately 2 months, so we believe that a follow-up period of at least 6 months is sufficient to assess radiographic alignment. However, 6 months is still too short. Follow-up of our patient cohort to 2-, 3-, or 5-year endpoints would more accurately demonstrate the durability of the procedure, particularly in terms of long-term pain relief, fusion status, and adjacent segmental lesion development. Follow-up will continue to track patients over time to provide additional and safer clinical data on the use of 3D printed materials. Third, all surgeries were performed by a single surgeon, and therefore differences in technique and experience cannot be accounted for. Fourth, our experience focuses only on lumbar fusion surgery; however, there may still be unrecognized biomechanical advantages and disadvantages when considering multilevel complex surgery. Finally, the use of 3D printing materials requires a high level of hospital platform. This study only included a single-center clinical study in our hospital, and it remains to be explored whether it is applicable to the application in other populations. It is expected that a joint multi-center study will be conducted to build a better platform for the promotion and application of 3D printing.

## 5. Conclusion

Posterior lumbar lamina decompression, bilateral pedicle screw fixation combined with 3D printed cage interbody fusion can obtain good clinical effect in the treatment of lumbar degenerative diseases. It has a good effect in improving clinical symptoms such as lumbar degeneration, lumbar and leg pain and numbness caused by nerve compression. And it is excellent in restoring the height of intervertebral space, the height and area of intervertebral foramen, the physiological curvature of lumbar vertebrae and the stability of lumbar vertebrae. 3D printed interbody fusion cage can be an ideal substitute for intervertebral bone grafting. The stable fusion time of interbody fusion cage after lumbar fusion is mostly from 3 months to half a year after operation and it may shift in the intervertebral space or even protrude into the spinal canal in the early stage of fusion.

## Author contributions

**Conceptualization:** Zi Wang.

**Data curation:** Dongzhe Zhang, Zepei Zhang.

**Formal analysis:** Zi Wang.

**Funding acquisition:** Jun Miao.

**Writing – original draft:** Zi Wang.

**Writing – review & editing:** Jun Miao.

## Supplementary Material

**Figure:**